# Metabolic and Functional Evaluation of the Heart and Lungs in Pulmonary Hypertension by Gated 2-[18F]-Fluoro-2-deoxy-D-glucose Positron Emission Tomography

**DOI:** 10.1177/2045893217701917

**Published:** 2017-03-10

**Authors:** Didem Saygin, Kristin B. Highland, Samar Farha, Margaret Park, Jacqueline Sharp, Emir Charles Roach, W.H. Wilson Tang, James D. Thomas, Serpil C. Erzurum, Donald R. Neumann, Frank P. DiFilippo

**Affiliations:** 1 Department of Pathobiology, Lerner Research Institute, Cleveland Clinic, Cleveland, OH, USA; 2 Respiratory Institute, Cleveland Clinic, Cleveland, OH, USA; 3 Department of Cardiovascular Medicine, Heart and Vascular Institute, Cleveland Clinic, Cleveland, OH, USA; 4 Heart and Vascular Institute, Northwestern University Hospital, Chicago, IL, USA; 5 Department of Nuclear Medicine, Cleveland Clinic, Cleveland, OH, USA

**Keywords:** pulmonary hypertension, positron emission tomography, PET, cardiac PET, lung PET

## Abstract

Pulmonary hypertension (PH) is associated with a metabolic shift towards glycolysis in both the right ventricle and lung. This results in increased glucose uptake to compensate for the lower energy yield of glycolysis, which creates a potential for 2-[18F] fluoro-2-deoxy-D-glucose (FDG) positron emission tomography (PET) to be a useful tool in the evaluation of participants with PH. We investigated the utility of PET for PH by comparing FDG-PET uptake in the right ventricle and lungs in 30 participants with PH and eight healthy controls and correlating these measurements with echocardiographic (ECHO) measurements and other traditional assessments commonly used in PH. All participants underwent gated FDG-PET scanning in the fasting state, ECHO, six-minute walk test (6MWT), and blood draw for NT-proBNP. Participants also completed the CAMPHOR questionnaire. Right ventricular (RV) end-diastolic and end-systolic volumes, RV ejection fraction, and FDG uptake by PET were significantly different between PH and healthy controls and strongly correlated with plasma NT-proBNP levels and RV ECHO parameters including TAPSE, RV systolic pressure, Tei index, and global peak systolic strain. In addition, lung standardized uptake value (SUV) was also found to be significantly higher in participants with PH than healthy controls. However, lung SUV did not show any significant correlations with NT-proBNP levels, 6MWT, or functional and pressure measurements by ECHO. In this study, we demonstrated the ability to evaluate both lung and right heart metabolism and function in PH by using a single gated FDG-PET scan.

Pulmonary hypertension (PH) results in abnormalities of right ventricular (RV) morphology and function as a direct consequence of pressure and volume overload of the right ventricle. Changes in RV morphology include hypertrophy, increased end-diastolic muscle fiber length, and dilation. These changes are associated with decreased RV capillary density and decreased right coronary artery perfusion pressure (associated with increased RV systolic pressure and RV mass) making the RV myocardium more vulnerable to ischemia.^1,2^ Under ischemic conditions, the myocardium shifts from oxidative phosphorylation to anaerobic glycolysis. This requires increased glucose uptake to compensate for the lower energy yield of glycolysis. In PH, there is also increasing evidence for a pathologic metabolic shift to glycolysis in the absence of ischemia in both the lungs and heart.^3–11^ This metabolic shift to a glycolytic pathway creates further potential for 2-[18F] fluoro-2-deoxy-D-glucose (FDG) positron emission tomography (PET) to be a useful tool in the evaluation of RV metabolism and function and pulmonary vascular changes seen in PH.

Using gated FDG-PET, we performed metabolic and functional imaging of the heart and metabolic imaging of the lungs in participants with PH and healthy controls. We correlated quantitative PET measurements with echocardiographic (ECHO) measurements and other traditional assessments commonly used in PH.

## Materials and methods

### Study population

This study was approved by the Cleveland Clinic Foundation Institutional Review Board, and written informed consent was obtained from all participants. At least 30 individuals with PH were targeted to enroll because of the pilot nature of this study. The diagnosis of PH was confirmed by historical right heart catheterization. Participants were required to have a resting mean pulmonary artery pressure (mPAP) ≥25 mmHg and a pulmonary artery occlusion pressure (PAOP) ≤12 mmHg. Individuals with World Heath Organization group 2 PH (secondary to left heart disease) were excluded from the study. All participants underwent transthoracic echocardiography, a gated FDG-PET scan, six-minute walk test (6MWT), and blood draw for N-Terminal pro-brain natriuretic peptide (NT-proBNP) (Roche Cobas e411, Roche Diagnostics, IN, USA; range 5–35,000 pg/mL, intra- and inter-assay coefficient of variability are 1.3–4.2% and 1.8–4.6%, respectively). Each individual also answered the Cambridge Pulmonary Hypertension Outcome Review (CAMPHOR) questionnaire. All clinical, imaging, and laboratory data were obtained on the same day for each individual. Healthy controls undergoing FDG-PET scans were volunteers without history of chronic disease or medication use.

### Gated FDG-PET

PET metabolic images were acquired with the individuals in the fasting state for a minimum of 8 h. A 370 MBq (10 mCi) dose of 18F-FDG was administered intravenously. Data were acquired on a PET/CT scanner (Biograph mCT, Siemens Molecular Imaging, Hoffman Estates, IL, USA) after a prescribed 90-min uptake period prior to imaging (mean ± SD = 94 ± 8 min; range = 77–119 min). The 22-cm axial field of view was positioned to include the heart and most of the lungs. A low-dose CT scan was acquired (120 kVp, 11 mAs, 4 mm slice thickness, 1.0 pitch) for the purpose of PET attenuation correction. During the CT scan, the individual was instructed to hold his/her breath at near-end expiration in order to optimize anatomic matching with the free-breathing PET scan. PET data were then acquired for 15 min in list mode along with the ECG-gating signal. An initial static image was reconstructed to assess image registration and to allow for adjustment of CT alignment, if necessary. After verifying CT alignment, static PET images and eight-frame gated PET images were reconstructed as follows: iterative OS-EM algorithm with time-of-flight and resolution modeling (Ultra-TrueX) three iterations, 21 subsets, 6-mm Gaussian post-filter, 128 × 128 matrix, 1.8 zoom.

**Fig. 1.**
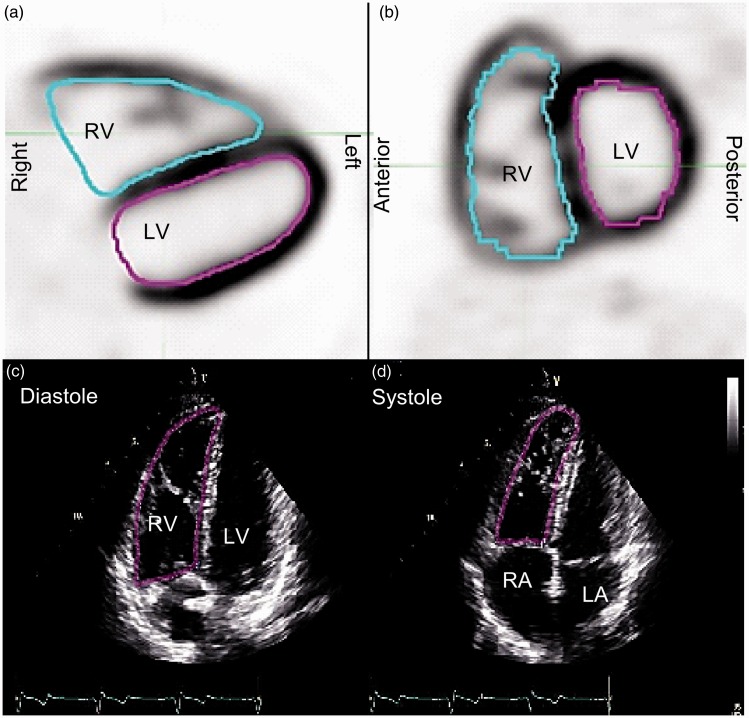
Illustration of contours drawn to measure ventricular volumes. PET trans-axial (a) and sagittal (b) slices are shown with the RV contour (blue) and the LV contour (magenta). RV contours are shown in magenta on four chamber ECHO images at end diastole (c) and end systole (d). PET contours are drawn in a range of trans-axial slices to form a 3D volume, whereas an ECHO area contour is drawn in a single 2D image. RV = right ventricle, LV = left ventricle.

End-diastolic (ED) and end-systolic (ES) PET images were extracted from the gated PET dataset by summing frames 1 and 8 for ED and frames 4 and 5 for ES Images. Summing the frames improved image quality and improved the ability to determine the myocardial boundary in many cases. The inner surface of the RV volumes were delineated manually on ED and ES images using the 3D Brush contouring tool of an image processing workstation (MIM version 6.5, MIM Software, Cleveland, OH, USA), allowing for calculation of the RV ejection fraction (RVEF) (Fig. 1).

Fused PET/CT images were analyzed quantitatively by a single-blinded radiologist (DRN). PET/CT scan overlay facilitated accurate drawing and positioning of regions of interest on static cardiac FDG-PET images for analysis of standardized uptake value (SUV) in the left ventricular (LV) free wall, interventricular septum, RV free wall, and right atrial (RA) free wall. Maximum PET SUV was recorded in the RV, RA, and LV free walls and the interventricular septum. RV/LV SUV ratio was calculated from these measurements.

In order to assess lung FDG uptake, a total of 24 regions per individual were analyzed, located in the upper/mid/lower levels and anterior/posterior regions of each lung. Mean CT Hounsfield Units (HU) and mean PET SUV were recorded for each region. Mean SUV of the lung was calculated as the average over these 24 regions. Lung FDG uptake was quantified in three ways: (1) SUV_M_ was the measured SUV, without concern for the various lung components; (2) SUV_L_ was the SUV corrected for the volume fraction of air only, which represents the mean uptake in bulk lung material, calculated according to SUV_L_ = SUV_M_/(1 + HU/1000); (3) SUV_T_ was the SUV corrected for the volume fractions of air and blood, which represents the mean FDG uptake in lung tissue (all that is not air or blood). The average blood fraction in lung (lung blood volume/total lung volume) was estimated to be 16% based on previously reported measurements in dogs.^12,13^ SUV_T_ was then calculated according to SUV_T_ = (SUV_M_ – 0.16 SUV_B_)/(1 + HU/1000 – 0.16), where SUV_B_ was the blood pool SUV measured as the mean SUV of the LA cavity and the descending thoracic aorta. When drawing regions, caution was taken to avoid including adjacent structures that would have affected quantitative results. For example, regions outlined in the lung did not include large blood vessels, and regions drawn for blood pool measurements only included the central portion of the cavity.

### ECHO

A comprehensive transthoracic Doppler echocardiogram (Vivid 9, GE Healthcare, Horten, Norway) was performed by a single registered advanced cardiac sonographer (MP) and included standard two-dimensional (2D) imaging, m-Mode, tissue and spectral Doppler for assessment of RV size, function, and right heart hemodynamics. All measurements were averaged over three cardiac cycles. Heart rate was calculated from an average of three R-R intervals. Blood pressure, height, and weight were recorded just prior to imaging. RV lateral annular pulsed wave tissue Doppler imaging was recorded from the basal segment. Annular peak systolic velocity (S’) served as a measure of RV function.

The following right heart functional parameters were analyzed from an apical four-chamber view modified to focus on the RV per the American Society of Echocardiography (ASE) guidelines.^14,15^ RV end-diastolic (RVED) and RV end-systolic (RVES) basal diameters were measured at the level of the tricuspid annulus. RV fractional shortening was calculated from RVED and RVES areas using the formula (RVED area - RVES area)/RVED area ×100%. Tricuspid annular plane systolic excursion (TAPSE) was calculated using m-mode echocardiography with cursor placement parallel to the RV lateral free wall. Right atrial volumes were measured at peak systole when the RA was largest in size. RA pressure (RAP) was estimated based on inferior vena cava size and compressibility with normal respiration and during sniff testing per ASE guidelines.^14,15^ Pulsed wave Doppler flow of the hepatic veins was evaluated for systolic reversal. RV wall thickness was measured in the subcostal window apical four-chamber view during end-diastole at the TV annular level. If the subcostal window was not adequate, the apical four-chamber view was used.

The tricuspid valve (TV) was interrogated for flow abnormalities with color flow and spectral Doppler with particular attention focused on the TV regurgitation (TR) peak velocity recorded from multiple imaging sites. Images were enhanced with agitated saline contrast as needed to optimize measurement of peak TR velocity. RV systolic pressure (RVSP) was calculated using the peak TR velocity (V) and the simplified Bernoulli equation, RVSP = 4V^2^ plus estimated RAP in the absence of pulmonic stenosis and RV outflow tract (RVOT) obstruction.

From the parasternal window, proximal RVOT linear dimensions were measured at the level of the pulmonic valve (PV) annulus. Pulsed wave Doppler was measured just proximal to the PV, with the closure snap clearly identified to derive the RVOT time velocity integral (TVI) and RV ejection time. RV Tei index or the RV index of myocardial performance (RIMP) was calculated as TV closing to opening time minus RV ejection time, divided by the RV ejection time. Pulmonary vascular resistance (PVR) was derived by the equation TRV/TVI (RVOT) × 10 + 0.16.

From the apical four-chamber window, 2D speckle tracking images of the LV and RV (RV focused view) were obtained at a frame rate of 50–70 frames/s (fps). RV longitudinal global peak systolic strain (RVPSS) was analyzed offline using Echo Pac Clinical Workstation Software (General Electric, Milwaukee, WI, USA). RV global longitudinal strain was calculated using three lateral RV wall segments and three inter-ventricular septum segments.

### Six-minute walk test, CAMPHOR questionnaire, and plasma NT-proBNP levels

All participants completed a 6MWT that was conducted according to ATS guidelines. Heart rate recovery was measured as difference between heart rate at 6 min and 1 min after completion of the walk. Participants completed the PH-health related quality of life CAMPHOR questionnaire. Individuals also had blood drawn to determine plasma NT-proBNP levels, which were determined by electrochemiluminescence sandwich immunoassay.

### Statistical analysis

Student t-test or pooled t-test was used to compare hemodynamic and imaging parameters between the individuals with PH and healthy controls where appropriate. Spearman correlation was calculated for RVED, RVES volumes, RVEF, and pulmonary and cardiac FDG uptake values versus clinical, laboratory, and ECHO parameters. Intra-rater variability was assessed with the Bland–Altman method. Statistical significance was accepted at a level of *P* = 0.05. A correction for multiple comparisons was not performed since this was a pilot study with a small sample size.

## Results

We evaluated 30 individuals with PH and eight healthy controls that were matched by age, gender, smoking status, and race (Table 1). Blood glucose levels were similar between groups (*P* = 0.2, 95% confidence interval [CI] = –4.5 to 19.9) (Table 1). Of the 30 individuals with PH, most had heritable (n = 12) or idiopathic forms (n = 9), while others had PH associated with interstitial lung disease (n = 2), chronic thromboembolic (n = 2), scleroderma (n = 2), uncorrected congenital systemic-to-pulmonary shunt (n = 1), hereditary spherocytosis (n = 1), and hemochromatosis (n = 1).

**Table 1.** Characteristics and PET measurements of the right ventricle and lung in individuals with PH vs. healthy controls.

	PH (n = 30)	Controls (n = 8)	*P* value
Age (years)	44 ± 12	42 ± 12	0.7
Gender (M/F)	9/21	1/7	0.6
Smoking status (no/ex-smoker/smoker)	21/9/0	6/2/0	1
Race (Caucasian/ African American/ Others)	23/6/1	7/1/0	0.7
Blood glucose levels (mg/dL)	88 ± 15	80 ± 13	0.2
Distance achieved in 6MWT (feet)	1510 ± 380		
Heart rate recovery (bpm)	18 ± 27		
CAMPHOR total score	19 ± 14		
NT-proBNP levels (pg/mL)	90 ± 472		
NYHA Class (I/II/III)	5/19/6		

Numerical variables with parametric distribution are presented as mean ± standard deviation; numerical variables with non-parametric distribution are presented as median ± interquartile range.

6MWT = six-minute walk test.

### FDG-PET SUV measurements

Intra-rater agreement was evaluated for SUV measurements and showed almost no mean difference (difference mean −0.059 and 95% limits of agreement of 0.006 and −0.126) with high correlation coefficient (0.992).

### Pulmonary FDG Uptake

There was no statistically significant difference in FDG uptake in the 12 different lung regions for each lung (*P* = 0.54), between the right and left lung (*P* = 0.23), or between the upper and lower lobes of each lung (*P* = 0.17). Thus, we calculated the average SUV by including all levels of both lungs.

Average lung SUV_M_ (not corrected for air or blood components) of individuals with PH (0.50 ± 0.15) was significantly higher than in healthy controls (0.37 ± 0.09; *P* = 0.01) (Table 2). When the lower lobes of both lungs were excluded due to the potential effect of scatter from liver on the right side and heart on the left side, the difference between individuals with PH and healthy controls was slightly more significant (*P* = 0.008). On the other hand, SUV_L_ (corrected for air component only) of PH and healthy controls was not significantly different (*P* = 0.10) (Table 2), and this result did not change with exclusion of the lower lobes (*P* = 0.15). SUV_T_ (corrected for air and blood components) of all lung segments was significantly higher in individuals with PH than healthy controls (*P* = 0.03) (Table 2). Exclusion of the lower lobes changed the significance of the difference between groups (*P* = 0.056). Because of the heterogeneity of our study population, we also performed a subanalysis comparing lung SUV_T_ of the patients with idiopathic and/or hereditary PAH with healthy controls and found that SUV_T_ was significantly higher in patients with idiopathic (n = 9) and hereditary (n = 12) PH (2.04 ± 0.73) than healthy controls (1.59 ± 0.38) (*P* = 0.04). In addition, comparison between idiopathic (2.13 ± 0.63) and hereditary PAH (1.98 ± 0.81) cases did not show significant difference (*P* = 0.6).

**Table 2.** PET measurements of the right ventricle and lung in individuals with PH vs. healthy controls.

	PH (n = 30)	Controls (n = 8)	*P* value
Heart			
RV end-diastolic volume (cm^3^)	145 ± 85	63 ± 16	<0.0001
RV end-systolic volume (cm^3^)	96 ± 79	43 ± 10	<0.0001
RV ejection fraction (%)	39 ± 13	30 ± 5	0.04
RV stroke volume (cm^3^)	49 ± 17	20 ± 7	0.0002
RV/LV SUV ratio	1.1 ± 0.9	0.3 ± 0.2	0.03
Lung			
SUV_M_ (g/mL)	0.50 ± 0.15	0.37 ± 0.09	0.01
SUV_L_ (g/mL)	1.61 ± 0.31	1.41 ± 0.27	0.10
SUV_T_ (g/mL)	2.00 ± 0.65	1.59 ± 0.38	0.03

Numerical variables with parametric distribution are presented as mean ± standard deviation; numerical variables with non-parametric distribution are presented as median ± interquartile range.

RV = right ventricle, LV = left ventricle, SUV_M_ = measured standardized uptake value, SUV_L_ = SUV corrected for air component, SUV_T_ = SUV corrected for air and blood components.

Distance achieved during the 6MWT did not correlate with any of the pulmonary SUV measures: average lung SUV_M_ (r = –0.3, *P* = 0.07), SUV_L_ (r = −0.2, *P* = 0.1), and SUV_T_ (r = −0.2, *P* = 0.3). Similarly, heart rate recovery did not correlate with average lung SUV_M_ (r = 0.09, *P* = 0.6), SUV_L_ (r = 0.08, *P* = 0.7), or SUV_T_ (r = −0.1, *P* = 0.5). CAMPHOR total score was not correlated with average lung SUV_M_ (r = 0.1, *P* = 0.4), SUV_L_ (r = 0.004, *P* = 0.9), or SUV_T_ (r = 0.03, *P* = 0.9). NT-proBNP levels also did not correlate with average lung SUV_M_ (r = −0.09, *P* = 0.6), SUV_L_ (r = −0.1, *P* = 0.2), or SUV_T_ (r = −0.2, *P* = 0.4).

### Cardiac FDG uptake

**Fig. 2.**
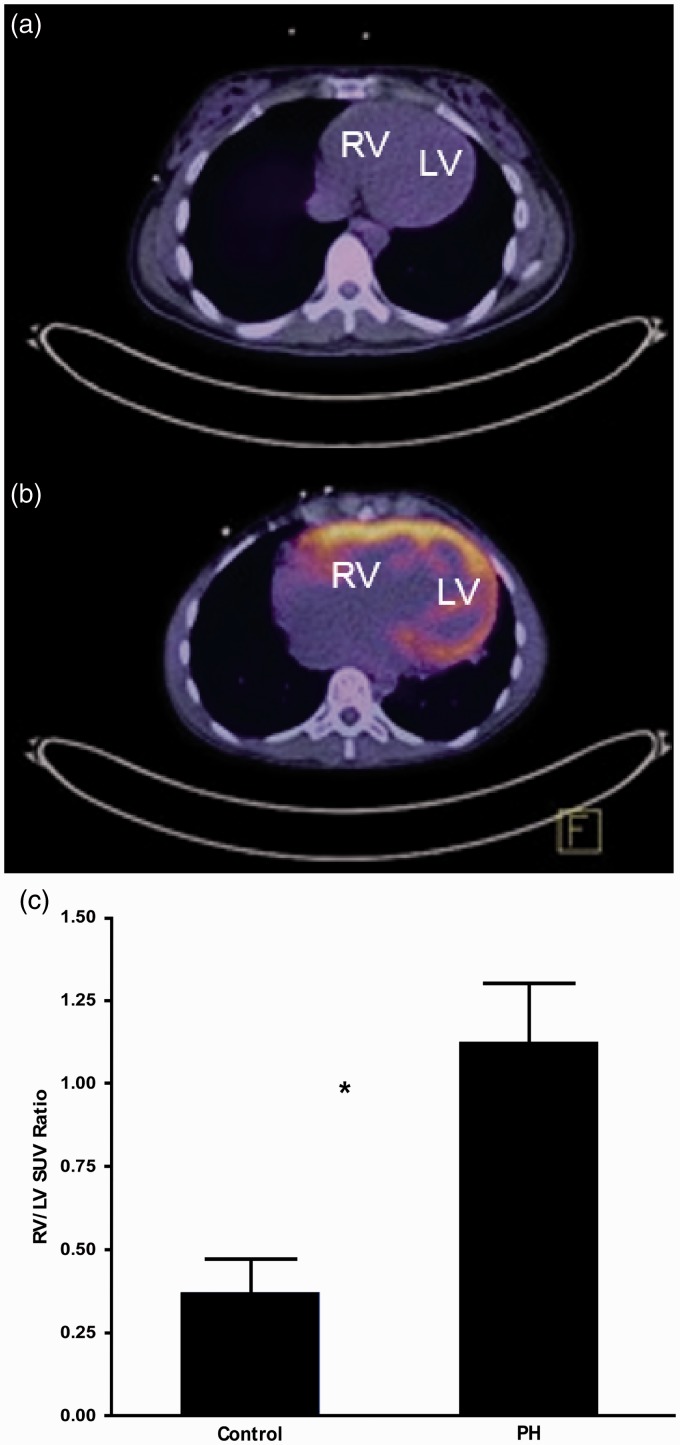
Fused PET/CT trans-axial slices of healthy control (a) and PH (B) participants. The control has minimal FDG uptake in the myocardium, whereas the PH participant has significant uptake, especially in the right ventricle. RV/LV SUV ratio in healthy controls and individuals with PH (c). Data are represented as mean ± standard error of the mean. Asterisk indicates statistically significant difference (*P* = 0.03). RV = right ventricle, LV = left ventricle, SUV = standardized uptake value.

**Fig. 3.**
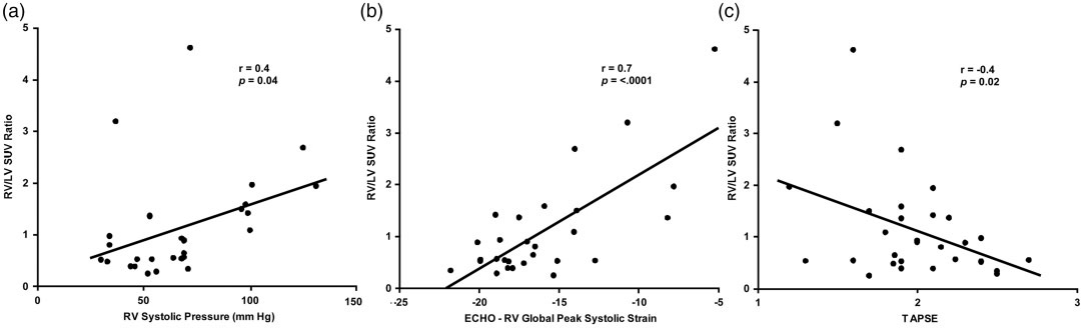
RV/LV SUV ratio is related to RV function and pressure measurements by ECHO. RV = right ventricle, LV = left ventricle, TAPSE = tricuspid annular plane systolic excursion.

Individuals with PH had significantly higher RV/LV SUV ratios compared with healthy controls (Fig. 2; Table 2). A subanalysis comparing idiopathic/hereditary PH (1.37 ± 1.05) and healthy controls (0.37 ± 0.28) revealed a more significant result (*P* = 0.0006). Also, comparison between idiopathic (1.20 ± 0.75) and hereditary PAH (1.49 ± 1.23) cases did not show a significant difference (*P* = 0.5). RV/LV SUV ratio significantly correlated with RA volume and RAP, RVES and RVED areas, RVES and RVED diameters, RV fractional shortening, RV systolic pressure (RVSP), TAPSE, and RV global peak systolic strain, whereas it did not correlate with RV thickness, RV Tei index, and S′ (Table 3; Fig. 3).

**Table 3.** Correlation between PET cardiac measurements vs. ECHO measurements.

ECHO	PET									
	RVED volume (cm^3^)		RVES volume (cm^3^)		RVEF (%)		RV stroke volume (cm^3^)		RV/LV SUV ratio	
	R	*P* value	R	*P* value	R	*P* value	R	*P* value	R	*P* value
Right atrial volume (mL)	0.6	0.001	0.5	0.001	−0.4	0.01	0.3	0.1	0.4	0.01
Right atrial pressure (mmHg)	0.4	0.04	0.3	0.07	−0.2	0.2	0.3	0.06	0.5	0.002
RVES area (cm^2^)	0.8	<0.0001	0.9	<0.0001	−0.8	<0.0001	0.09	0.6	0.7	<0.0001
RVED area (cm^2^)	0.8	<0.0001	0.8	<0.0001	−0.7	<0.0001	0.1	0.3	0.6	<0.0001
RVES diameter (cm)	0.5	0.002	0.6	0.001	−0.5	0.002	0.09	0.6	0.4	0.01
RVED diameter (cm)	0.8	<0.0001	0.8	<0.0001	−0.7	<0.0001	0.1	0.3	0.6	<0.0001
RV fractional shortening (%)	−0.4	0.01	−0.5	0.006	0.6	0.0007	0.07	0.7	−0.5	0.001
RV Tei index	0.4	0.01	0.5	0.007	−0.6	0.0008	0.0008	0.9	0.2	0.1
RV thickness (cm)	−0.03	0.8	0.08	0.6	−0.3	0.1	−0.3	0.1	0.3	0.1
RV systolic pressure (mmHg)	0.6	0.001	0.6	0.001	−0.5	0.003	0.2	0.2	0.3	0.04
TAPSE (cm)	−0.3	0.1	−0.3	0.07	0.6	0.0008	0.1	0.3	−0.4	0.02
RV global peak systolic strain (%)	0.7	<0.0001	0.8	<0.0001	−0.8	<0.0001	−0.07	0.7	0.7	<0.0001
S′ (cm/s)	−0.4	0.01	−0.5	0.006	0.4	0.02	0.04	0.8	−0.3	0.09
PVR (Wood units)	0.5	0.009	0.5	0.008	−0.4	0.01	−0.1	0.6	0.3	0.051

RVES = right ventricular end-systolic, RVED = right ventricular end-diastolic, TAPSE = tricuspid annular plane systolic excursion, S′ = annular peak systolic velocity, PVR = pulmonary vascular resistance.

RV/LV SUV ratio also failed to correlate with six-minute walking distance (6MWD) (R = 0.1, *P* = 0.5), heart rate recovery (R = −0.2, *P* = 0.3), or total score on the CAMPHOR questionnaire (R = −0.2, *P* = 0.1). RV/LV SUV ratio also did correlate significantly with plasma NT-proBNP levels (Table 4).

**Table 4.** Correlation between PET cardiac measurements vs. plasma NT-proBNP levels.

	Plasma NT- proBNP levels (pg/mL)	
	R	*P* value
RV end-diastolic volume (cm^3^)	0.7	<0.0001
RV end-systolic volume (cm^3^)	0.7	<0.0001
RV stroke volume (cm^3^)	0.3	0.09
RV ejection fraction (%)	−0.4	0.02
RV/LV SUV ratio	0.5	0.004

RV = right ventricle, LV = left ventricle, SUV = standardized uptake value.

### Cardiac functional measurements

The ability to draw the RV contours depended on the degree of FDG uptake in the myocardium (Fig. 1); higher uptake facilitated the contouring with better visualization of the inner surface of the myocardium. In this study, 24 out of 30 FDG-PET scans of PH patients (80%) and 5 of 8 FDG-PET scans of controls (63%) had sufficient uptake to visually determine wall boundaries.

RVED, RVES volumes, RVEF, and RV stroke volume measured from gated FDG-PET images were all significantly higher in individuals with PH compared with healthy controls (Table 2). However, fasting PET images of healthy controls showed minimal uptake in the RV, which limited the ability to draw contours and to measure RVEF accurately. A subanalysis comparing idiopathic/hereditary PH and healthy controls yielded similar results (*P* = 0.001, 0.006, 0.008, 0.8 for RVED, RVES and RV stroke volumes, and RVEF, respectively) except RVEF. Also, a comparison between idiopathic and hereditary PAH cases did not show a significant difference (*P* = 0.82. 0.99, 0.21, 0.87 for RVED, RVES, and RV stroke volumes and RVEF, respectively).

**Fig. 4.**
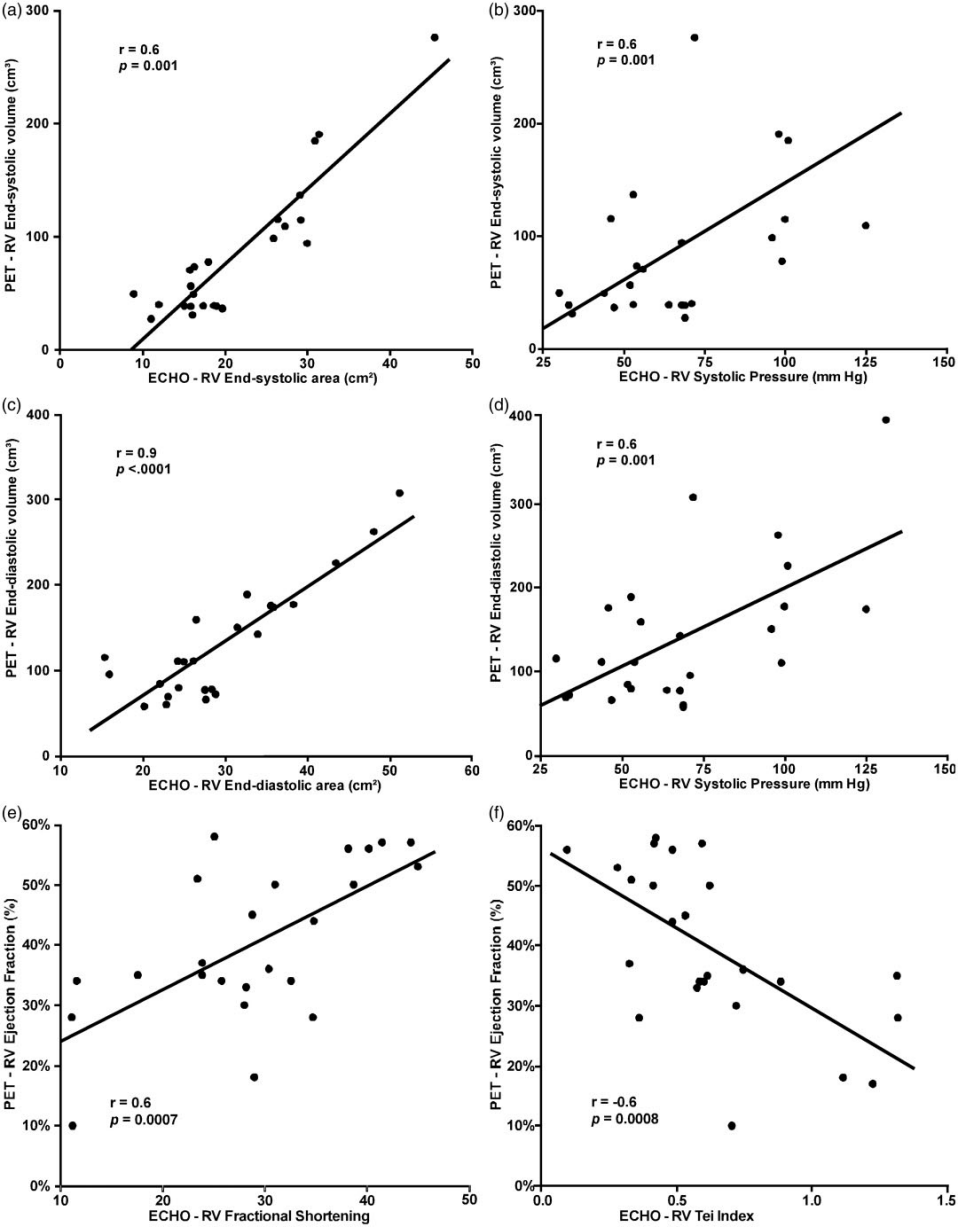
3D measurements of RV systolic volume (a, b), RV diastolic volume (c, d) and ejection fraction (e, f) are related to 2D measurements by ECHO.

PET RVED and RVES volumes significantly correlated with a number of ECHO parameters including: RA volume, RVES and RVED areas, RVES and RVED diameters, RV fractional shortening, RV Tei index, RVSP, RV global peak systolic strain, S′, and PVR. Strain values were not derived in two individuals due to technical complications in data transfer for one and poor technical quality of the RV image for the other. A correlation was not found with RV thickness and TAPSE (Table 3). PET RVEF correlated significantly with RA volume, RVES and RVED areas, RVES and RVED diameters, RV fractional shortening, RV Tei index, RVSP, TAPSE, RV global peak systolic strain, S′, and PVR (Table 3; Fig. 4) as measured by echocardiogram. PET RV stroke volume, however, did not show significant correlation with any RA or RV ECHO parameters (Table 3).

PET-derived RVED and RVES volumes, RVEF, and RV stroke volume were not significantly correlated with 6MWD (*P* = 0.6, 0.5, 0.2, 0.6, respectively), heart rate recovery (*P* = 0.9, 0.9, 0.4, 0.6, respectively), or CAMPHOR total score (*P* = 0.6, 0.4, 0.09, and 0.7, respectively). However, RVED and RVES volumes and RVEF significantly correlated with plasma NT-proBNP levels, whereas RV stroke volume did not (Table 4). NT-proBNP continued to have a significant correlation with RVED and RVES volumes, and RV ejection fraction even when two unusually high NT-proBNP levels were removed from the analysis (*P* = 0.001, 0.0004, 0.03, respectively).

Of note, plasma NT-proBNP level did not correlate with a number of echocardiographic features including: RVOT ejection time (r = −0.1, *P* = 0.4), RA volume (r = 0.3, *P* = 0.09), RVED diameter (r = 0.1, *P* = 0.3), RV Tei Index (r = 0.1, *P* = 0.3), RV thickness (r = 0.1, *P* = 0.3), right atrial pressure (r = 0.3, *P* = 0.057), TAPSE (r = −0.2, *P* = 0.1), and RV global peak systolic strain (r = 0.2, *P* = 0.1). Nevertheless, there was a significant correlation with RV systolic pressure (r = 0.4, *P* = 0.01).

## Discussion

Using a single gated FDG-PET scan, we demonstrated the ability to evaluate both lung metabolism and right heart metabolism and function in PH. The heart and most of the lungs were contained in the field of view of the PET/CT scanner, allowing for both organs to be imaged simultaneously while acquiring an electrocardiograph signal. The gated cardiac PET images provided a feasible and accurate measure of right heart function, as evidenced by comparison with ECHO measurements.

Evaluation of both lungs and heart with a single FDG-PET scan required us to make a choice between fasting versus glucose loading protocols. In the fasting state, the myocardium uses fatty acid as its primary energy source. In order to optimize image quality, a myocardial FDG-PET scan is typically performed after glucose loading, which results in increased insulin-driven glucose uptake and enhanced visualization of the myocardium. On the other hand, FDG-PET for cancer staging is performed in a fasting state, which leads to lower blood glucose levels to compete with FDG, enables cells to capture FDG independent from hormonal influence, and therefore better reflects the true glycolytic metabolic status of cells. Prior FDG-PET studies have demonstrated a glycolytic shift in both the RV and lungs of patients with PH.^3,4,8,10,11^ Since we aimed to investigate the glycolytic status of both the lung and heart, we focused on the applicability of fasting FDG-PET in the evaluation of RV and pulmonary glucose metabolism in PH.

An underlying goal of our approach was to utilize a PET scan protocol that is feasible to acquire in many clinical laboratories. Much progress has been made toward absolute quantification of lung PET images with kinetic modeling.^12^ Kinetic approaches require the continuous acquisition of dynamic PET data starting from the time of injection, typically for a duration of 1 h or longer. Accurate knowledge of the arterial input function (AIF) is critical to the success of kinetic modeling. Typically, the AIF is obtained by repeated sampling of arterial blood and measurement of radioactivity with correction for metabolites. A desirable alternative is image-based measurement of AIF, which greatly simplifies the procedure; however, use of an image-based AIF may cause errors that significantly affect kinetic analysis. For example, in their study comparing pulmonary FDG uptake in IPAH, Ruiter et al. acquired dynamic images with the intent of measuring metabolic rate of glucose uptake by Patlak analysis using an image-based AIF, but they obtained negative results and had to abandon the dynamic data.^16^ Our simplified protocol uses static image quantification with 90-min uptake period and with certain assumptions in the normalization for air and blood. Although less quantitative than full kinetic modeling with blood sampling, this approach requires significantly less resources and is much more easily implemented.

### Pulmonary FDG uptake

We previously demonstrated that there is a higher fasting FDG uptake in the lung of participants with PH compared with healthy controls.^8^ Following this finding, Marsboom et al. demonstrated that pulmonary FDG uptake increases with the earliest onset of PH in a monocrotaline rat model of PH and decreases after treatment with imatinib and dichloroacetate, two effective metabolic therapies for PH.^9^ We, and others, identified that this primarily occurs within the endothelium and vascular smooth muscle.^8–10^ Subsequently, the relationship between pulmonary FDG uptake and PH severity was investigated using both fasting and non-fasting PET scans.^11,16^ Ruiter et al. performed the first study investigating the correlation between pulmonary FDG uptake and PH severity, and no correlation was found between lung FDG uptake and 6MWD, mPAP, PVR, and plasma NT-proBNP levels, similar to our results.^16^ Although average lung SUV_M_ in controls agreed between their study and ours (0.37 versus 0.37), average lung SUV_M_ in PH participants was lower in their study compared with ours (0.41 versus 0.50, *P* = 0.08). Possible explanations include differences in uptake time prior to scanning (50 versus 90 min) and proportion of PH subtypes (IPAH versus heterogeneous) in study populations.

### RV/LV SUV ratio

RV failure is the major cause of mortality in individuals with PH. Therefore, non-invasive imaging techniques of the right ventricle are needed to help improve outcomes. A number of studies have explored the potential utility of FDG-PET uptake in the RV to improve diagnosis, management, and prognostication of participants with PH. In one of the first studies done in humans, Kluge et al. examined the relationship between RV/LV glucose uptake ratio and PH severity.^17^ Thirty individuals with PH underwent FDG-PET scans after oral glucose load and fatty acid inhibition. RV/LV FDG uptake ratio correlated with functional class and severity of PH as measured by Tei index; however, it did not correlate with PVR. In another study of interest, Can et al. compared RV/LV SUV ratio in participants with PH and healthy controls and examined its relationship with ECHO parameters and the 6MWT.^4^ The RV/LV SUV ratio was significantly higher in individuals with PH than healthy controls (23 individuals with PH, 16 controls) and was significantly correlated with pulmonary arterial systolic pressure, TAPSE, RV Tei index, and 6MWD. Oikawa et al. demonstrated that RV SUV was significantly correlated with mPAP, RAP, PVR, RV wall stress, and plasma BNP levels, but not with RV wall thickness and mass in 24 participants with PH.^18^ After three months of treatment with epoprostentol in ten of these participants, RV SUV was significantly decreased in those participants with a documented 30% or greater improvement in PVR. Furthermore, the percentage change of RV FDG uptake significantly correlated with improvement in PVR and RV systolic wall stress. Likewise, Fang et al. showed a significant improvement in RV/LV FDG uptake ratio after six months of treatment with sildenafil. Overall, these findings suggest that RV/LV SUV ratio may be a strong predictor of PH severity and may be useful to monitor response to treatment.^19^ Lastly, Tatebe et al. examined the relationship between RV/LV ratio and prognosis of PH.^20^ They included 27 individuals with PH who underwent gated FDG-PET scan using an oral glucose loading protocol, and followed them for 69 ± 49 months. They demonstrated that higher RV FDG update was associated with shorter time to clinical worsening and higher all-cause mortality, indicating that elevated RV SUV might be a potential prognostic marker in the management of PH.

Our study found that RV/LV FDG uptake ratio correlated with RA volume and RAP, RVES and RVED areas, RVED and RVES diameters, RV fractional shortening, RVSP, TAPSE, and RV global peak systolic strain. We did not find a correlation between RV/LV SUV ratio and 6MWD, CAMPHOR score, RV thickness, RV Tei index, and S′. These results are consistent with the literature as summarized above. An important distinguishing feature of our study is that unlike prior PET studies for PH performed under glucose load to maximize FDG uptake, the PET scans in our study were performed in the fasting state, which may account for some of the differences in our data. We demonstrated the feasibility of fasting FDG-PET in assessing RV metabolic activity, and our data suggest that it may allow for a deeper understanding of the metabolic mechanisms that lead to PH. Another distinguishing feature of our study is the use of gated PET acquisition to obtain functional cardiac measurements in addition to the metabolic evaluation of the lung and myocardium.

### RVED, RVES volumes, RV ejection fraction

Commercial software is available for automated cardiac PET analysis of LV regional myocardial perfusion, volume, EF, and wall motion. Wang et al. showed that by applying one such commercial software package to the RV instead of the LV, FDG-gated PET scan calculations of RVED and RVES volumes, and RVEF significantly correlated with similar measurements performed with cardiac MRI and cardiac CT.^21^ However, in that study, individuals were imaged under oral glucose load in order to increase myocardial FDG uptake and to facilitate automated identification of RV wall boundaries.

In our study, automated software failed to identify myocardial boundaries and manual processing was required. Our manual measurements of RV volumes and ejection fraction correlated significantly with RV echocardiographic parameters. A limitation of this technique is that the ability to determine myocardial wall boundaries from gated PET images depends highly on the degree of FDG uptake, which is lower in the fasting state compared with glucose-loaded state, as well as the level of image noise. Since each frame of an eight-frame gated PET image has one-eighth the counts as the corresponding static image, obtaining high quality ED and ES images can be challenging. For this study, we were fortunate to use a state-of-the-art PET scanner with an extended axial field of view, high count sensitivity, and time-of-flight capability. These features enabled the heart and lungs to be scanned simultaneously and with high image quality. Summing two of the eight frames improved ED and ES image quality. Even with improved count statistics from summing two frames, there were a few cases (6/30 PET scans) in which there was insufficient uptake above the noise level to determine myocardial wall boundaries in the ED and ES PET images. Considering that many PET scanners do not have time-of-flight capability and high count sensitivity, it is possible that myocardial wall determination might be more challenging or less accurate in some cases.

Although the difference in lung FDG uptake was small between PH participants and controls, it was sufficient to reach statistical significance in this population. However, lung SUV measurement does not appear to be sufficiently accurate to characterize PH severity in a single patient, and clinical use of lung SUV is unlikely. Cardiac RV/LV uptake ratio, on the other hand, showed much larger variation between PH participants and controls, and among PH participants themselves. Further study is needed to understand the underlying mechanisms. Potentially, differences or changes in RV/LV ratio may be sufficiently large to be useful in research studies or in a clinical environment. Functional parameters derived from cardiac PET images correlated strongly with ECHO parameters. ECHO’s simplicity, lower cost, and lack of ionizing radiation still make it most suitable for clinical use. RV functional parameters from three-dimensional (3D) PET may be potentially more accurate than 2D ECHO; however, this awaits further investigation with comparison to the gold standard, cardiac 3D MRI.

## Conclusions

In conclusion, gated FDG-PET offers both metabolic and functional evaluation of the heart and metabolic evaluation of the lungs for PH during a single imaging procedure. Lung SUV measurements were higher in PH than healthy controls, confirming prior results in the literature. Myocardial SUV measurements (RV/LV SUV ratio) strongly correlated with PH severity and may be useful for monitoring treatment response. Although imaging in the fasting state does not produce the highest myocardial uptake of FDG, there usually was sufficient uptake to allow for accurate 3D segmentation of the RV at ED and ES allowing for functional analysis. Our gated FDG-PET measurements of RV function correlated strongly with 2D ECHO measurements. Nevertheless, patient preparation is a topic for future research, as most published work on FDG imaging of the lung was done in the fasting state, whereas most FDG imaging of the heart was done in the glucose-loaded state.
